# Immune adaptor protein SKAP1 (SKAP-55) forms homodimers as mediated by the N-terminal region

**DOI:** 10.1186/s13104-018-3976-3

**Published:** 2018-12-06

**Authors:** Monika Raab, Klaus Strebhardt, Christopher E. Rudd

**Affiliations:** 1 Department of Obstetrics and Gynaecology, School of Medicine, J.W. Goethe-University, Theodor-Stern-Kai 7, 60590 Frankfurt, Germany; 2 German Cancer Consortium (DKTK)/German Cancer Research Center, Heidelberg, Germany; 3 Research Center-Maisonneuve-Rosemont Hospital (CRHMR), Montreal, QC H1T 2M4 Canada; 4 Cell Signalling Section, Department of Pathology, Cambridge University, Cambridge, CB2 1QP UK

**Keywords:** SKAP1, T-cells, Dimer, RapL

## Abstract

**Objective:**

Immune cell adaptor protein SKAP1 couples the antigen-receptor (TCR/CD3) with the activation of LFA-1 adhesion in T-cells. Previous work by ourselves and others have shown that SKAP1 can directly bind to other adaptors such as ADAP and RapL. However, it has been unclear whether SKAP1 can form homodimers with itself and the regions within SKAP1 that mediated homodimer formation.

**Results:**

Here, we show that SKAP1 and SKAP2 form homodimers in cells. Homodimer formation of immune adaptor protein SKAP1 (SKAP-55) are mediated by residues A17 to L21 in the SKAP1 N-terminal region. SKAP1 dimer formation was not needed for its binding to RapL. These data indicate that the pathway linking SKAP1 to RapL is not dependent on the homo-dimerization of SKAP1.

## Introduction

T-cells are activated by a combination of protein-tyrosine kinases and adaptor proteins which mediate the formation of multi-protein complexes [1, 2]. Immune cell adaptors regulate T-cell proliferation and function [1–3]. SLP-76 (SH2 domain containing leukocyte protein of 76 kDa) is one such adaptor that is needed for phospholipase Cγ1 (PLCγ1) activation, calcium mobilization and thymic differentiation [4, 5]. It has an N-terminal sterile-α motif (SAM) and a carboxy-terminal SH2 domain that binds to adhesion and degranulation-promoting adapter protein (ADAP) [6, 7] and the hematopoietic progenitor kinase-1 (HPK-1) [8]. The C-terminal SH2 domain SLP-76 binds to the ADAP [6, 7, 10], while ADAP in turn binds to SKAP-1 [10, 11]. SKAP-1 is an adaptor with a unique N terminus, a PH domain and a C terminal SH3 domain [9, 10]. SKAP1 SH3 domain binds to proline residues in ADAP while the ADAP-SH3-like domain binds to SKAP1 [13, 14]. SKAP1 couples the TCR to the activation of LFA-1 [11–17]. SKAP1 regulates RapL-Rap1 binding induced by antigen-receptor ligation [16–18]. SKAP55 dimer formation has been shown by imaging studies to stabilize SLP-76 micro-clusters and facilitate adhesion [19].

In this study, we have assessed whether SKAP1 and SKAP2 can form homodimers and the region involved in the dimerization. We show biochemically that SKAP1 and SKAP2 can form homodimers in the generation of signals in T-cells. Homodimer formation of immune adaptor protein SKAP1 (SKAP-55) are mediated by the N-terminal region.

## Main text

### Methods

#### Cell culture

293T cells were grown in DMEM culture medium with 10% fetal calf serum (FCS), 2 mM l-glutamine, penicillin, and streptomycin.

#### Antibodies

Antibodies to GFP and GST were from Santa Cruz. Anti-SKAP1 (BD Transduction Laboratories), anti-V5 (Invitrogen), anti-FLAG and anti-β-actin (Sigma) were purchased as assigned. HRP-conjugated secondary antibodies (1:5000) were from Santa Cruz and biotinylated secondary antibodies were from DAKO.

#### Constructs and transfection

The constructs of SKAP1 and SKAP2 were inserted into a pGEX5x-3 (GE Healthcare) and into a 3×Flag-tagged as well as the vectors encoding EGFP-tagged pcDNA3.1-Hygro (Invitrogen). Site-directed mutagenesis was conducted using QuickChange protocol and Pfu Ultra II Fusion HS DNA Polymerase (Stratagene). Transfections were conducted using BTX ECM 830 electroporator as described [18].

#### Immunoblotting

Precipitations were conducted by lysis in Triton X-100 lysis buffer followed by the incubation with antibody for 1–2 h at 4 °C and purification of complexes using protein G-Sepharose beads (10% w/v) as described [16–18]. For blotting, material on gels transferred onto nitrocellulose filters (Schleicher and Schuell) and detected using horseradish peroxidase-conjugated rabbit anti-mouse antibody together with enhanced chemiluminescence (ECL, Amersham Biosciences).

#### GST pull down assay

The expression of recombinant GST-proteins was induced in *Escherichia coli* BL21 cells at 37 °C for 2 h by the addition of 1 mM IPTG. GST-fused proteins were purified with the Cell Lytic B protocol (Sigma #B7435). Cell lysates were incubated with GST fusion proteins for 3 h followed by analysis in SDS-PAGE and western blotting as described [18].

### Results

#### SKAP1 binds to SKAP1 and SKAP2

**Fig. 1:**
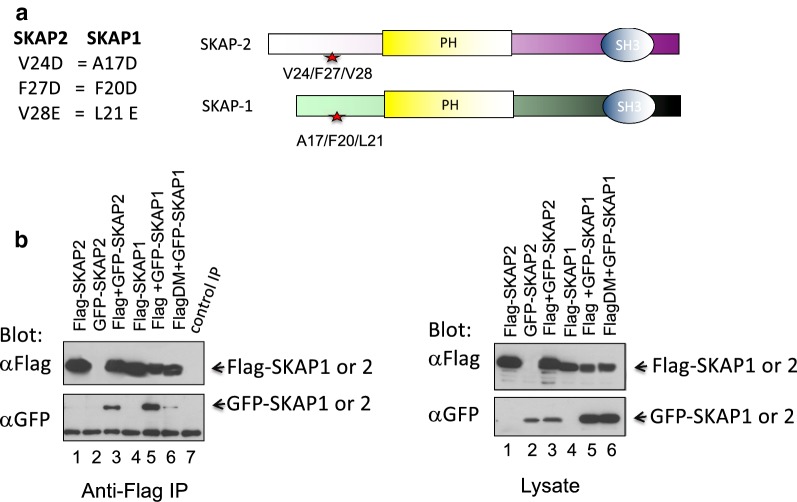
SKAP1 and SKAP2 form homodimers. **a** Model of the structure of SKAP1 and SKAP2. Mutations in the dimerization domain of SKAP-1 A17/F20/L21 is shown. **b** Co-precipitation of SKAP1 with SKAP-1 and SKAP2 with SKAP2. Left panel: anti-FLAG was used to precipitate antigen from lysates of transfected 293T cells followed by blotting with anti-FLAG or anti-GFP. Lane 1: Flag-SKAP2; lane 2: GFP-SKAP2; lane 3: Flag-SKAP2 and GFP-SKAP2; lane 4: Flag-SKAP1; lane 5: Flag and GFP-SKAP1; lane 6: FlagDM (A17/F20/L21) and GFP-SKAP1. Right panel: blotting of cell lysates from transfected 293T cells seen in left panel

To assess whether SKAP1 can interact with itself and SKAP2, each was expressed in 293T cells followed by precipitation with anti-Flag (Fig. 1). Cell lysates or precipitates were then blotted with anti-Flag or GFP. Flag-tagged SKAP1 and GFP-tagged SKAP1 were co-expressed followed by precipitation with anti-Flag. Anti-Flag precipitated Flag-tagged SKAP-1 as well as GFP-tagged SKAP1 from cell lysates (lane 5). This indicates that SKAP1 could form homodimers with itself. Similarly, Flag-tagged SKAP2 and GFP-tagged SKAP2 were co-expressed followed by precipitation with anti-Flag. Anti-Flag precipitated Flag-tagged SKAP2 as well as GFP-tagged SKAP2 from cell lysates (lane 3). This indicates that SKAP2 could form homodimers with itself.

To assess whether SKAP1 homodimer formation was dependent on the N-terminal domain, a version of Flag-tagged SKAP1 with mutations in residues A17/F20/L21 were co-expressed with GFP-tagged wild-type SKAP1 followed by anti-Flag co-precipitation. Mutation of residues A17/F20/L21 abrogated the homodimeric binding of SKAP1 with itself (lane 6). As a control, the blotting of cell lysates showed the expression of the various Flag and GFP tagged proteins (right panel). These data demonstrate the residues in the region of A17 to L21 of the N-terminal region of SKAP1 mediates dimer formation.

**Fig. 2:**
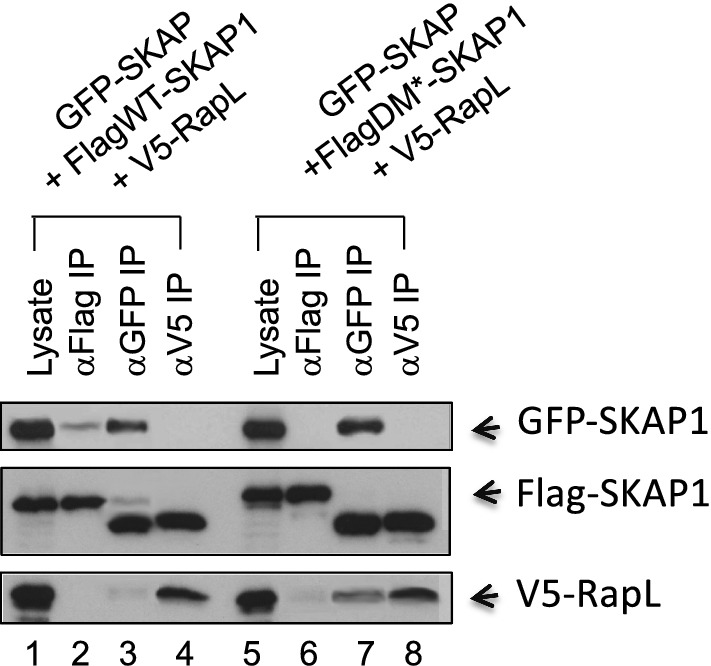
Dimerisation is not needed for SKAP1 binding to RapL. L293T cells transfected with SKAP-GFP + FlagWT-SKAP1 + V5-Rap (lanes 1–4) or SKAP-GFP + FLAGDM*-SKAP1 + V5-RapL (lanes 5–8). Lane 1,5: lysate; lane 2,6: anti-Flag IP; lane 3,7: anti-GFP IP; lane 4,8: anti-V5 IP

We previously showed that SKAP1 binds to RapL and is needed for RapL binding the GTPase Rap1 and the activation of LFA-1 adhesion. N-terminal SKAP1 domain binds to the C-terminal SARAH domain of Rap1. We also show that SKAP1 is needed for RapL binding to membranes in a manner dependent on the PH domain of SKAP1 and the PI3K pathway [16, 17]. Others have reported other components such as Rap1-dependent integrin regulator Rap1-GTP-interacting adaptor molecule (RIAM) in the multimeric complex [18]. We therefore next asked whether SKAP-1 monomer or dimer formation was needed for SKAP1 binding to RapL (Fig. 2). Tagged wild-type or A17/F20/L21 mutant SKAP1 was co-expressed with RapL in 293T cells and assessed for co-precipitation. While anti-Flag precipitated GFP-tagged SKAP1, the dimer failed to co-precipitate RapL (lane 2). Similarly, anti-GFP coprecipitated Flag-SKAP1 but a faint RapL band (lane 3). For unknown reasons, the Flag-tagged SKAP1 bound to GFP-SKAP1 consistently migrated at a lower Mr suggestive of a post-translational change in the protein. Intriguingly, anti-V5 precipitated V5-tagged RapL with only the lower Mr Flag-tagged SKAP1 (lane 4). This suggests that the lower Mr version of SKAP1 preferentially associates with RapL. Intriguingly, the same patterns of co-precipitation were observed Flag-A17/F20/L21 SKAP1 (lanes 7 and 8). Attempts were made to transfect primary T-cells for expression but were limited by the low levels of expression that precluded an analysis of binding in these cells. These data indicate that SKAP1 dimer formation is not needed for its binding to RapL.

**Fig. 3:**
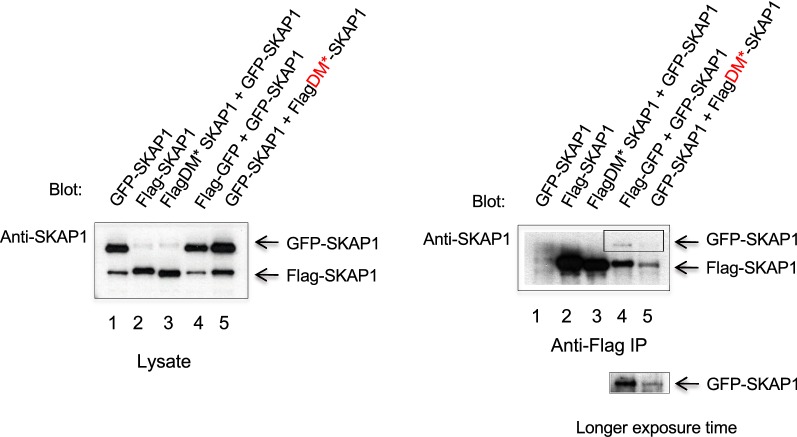
Confirmation of dimerisation with anti-SKAP1 blotting. L293T cells transfected with GFP-SKAP (lane 1), Flag-SKAP1 (lane 2), FlagDM*SKAP1 + Flag SKAP1 (lanes 3), Flag-SKAP1 and SKAP-GFP + (lanes 4) and GFP-SKAP1 + FlagDM*SKAP1 (lanes 5) (left panel). Right panel: anti-Flag precipitates from lysates in left panel. Lower insert shows a longer exposure of a section of lanes 4 and 5

Lastly, we next showed the presence of the SKAP1 dimer by blotting with anti-SKAP1 rather than antibodies to tags on the proteins (Fig. 3). Combinations of Flag and GFP-tagged SKAP1 were expressed in 293T cells (left panel) and subjected to precipitation using anti-Flag and the followed by blotting with anti-SKAP1 (right panel). Anti-Flag precipitation of Flag SKAP or FlagDM-Skap1 co-precipitated co-expressed GFP-SKAP1 as detected by anti-SKAP-1 (lanes 4 and 5; longer exposure below). We attempted to co-express these vectors in primary mouse T-cells from spleen but were unable to obtain sufficiently high levels of expression of both proteins to carry out similar analysis with these cells. These data confirmed that SKAP1 dimer formation as detected with anti-SKAP1.

### Discussion

Overall, our study shows that SKAP1 forms homodimers dependent on residues A17/F20/L21 in the N-terminus of SKAP1. We previously showed that this region has alternating leucine residues and shares homology with the coiled-coil domain of SKAP-2 [9]. Both SKAP1 and SKAP2 bind ADAP (FYB) through their SH3 domains and served as substrates for the FYN kinase in T cells [9, 10]. SKAP1 also colocalizes with another the ADAP binding protein, SLP-76. Mutation of the YDDV sites (termed M12) that disrupt SLP-76 SH2 domain binding interferes with ADAP binding and decreases conjugation and LFA-1 clustering [13]. SKAP1 Src homology 3 (SH3) domains also stabilizes SLP-76 micro-clusters [19].

By contrast, the N-terminal region of SKAP1 binds to RapL such that a RapL mutation (L224A) abrogates SKAP1 binding and arrests T-cells even in the absence of antigen [17]. We now extend these findings by showing that dimerization is not required for the direct binding of SKAP1 to RapL. In fact, the A17/F20/L21 mutant often bound more to RapL than did wild-type SKAP-1. In this context, it is possible that dimerization limits SKAP1 binding to RapL. This contrasts with the reported requirement for the SKAP dimerization for binding to RIAM [19]. It is possible that dimerization may act on functions that are distinct from SKAP1–RapL activation of LFA-1. In the same manner, while the expression of the related protein SKAP-55R failed to compensate for the loss of SKAP1 in LFA-1 clustering in mouse T-cell hybridomas [20], it appears to substitute for SKAP1 in stabilizing the formation of microclusters in Jukat T-cells [19]. The full range of functions mediated by SKAP1 dimerization remain to be demonstrated in future studies.

### Limitations

Work restricted to non-lymphoid cells.

## Abbreviations

SLP-76: SH2 domain containing leukocyte protein of 76 kDa
PLCγ1: phospholipase Cγ1 (PLCγ1)
SAM: sterile-α motif
ADAP: adhesion and degranulation-promoting adapter protein
HPK-1: hematopoietic progenitor kinase-1
